# *Pterygodermatites (Mesopectines) whartoni* (Nematoda: Rictulariidae) encysted larvae in invasive Cuban treefrogs (*Osteopilus septentrionalis*) from Florida, United States

**DOI:** 10.3389/fvets.2024.1353975

**Published:** 2024-05-10

**Authors:** Kelsey Lykins, Robert J. Ossiboff, Ellis Chase, Nina Thompson, Terence M. Farrell, Timothy Wu, Steve A. Johnson, Heather D. S. Walden

**Affiliations:** ^1^Department of Comparative, Diagnostic and Population Medicine, College of Veterinary Medicine, University of Florida, Gainesville, FL, United States; ^2^Department of Biology, Stetson University, DeLand, FL, United States; ^3^Department of Population Medicine, Section of Anatomic Pathology, College of Veterinary Medicine, Cornell University, Ithaca, NY, United States; ^4^Department of Wildlife Ecology and Conservation, University of Florida, Gainesville, FL, United States

**Keywords:** paratenic, encysted larvae, amphibian host, nematode, helminth

## Abstract

Species of *Pterygodermatites* are spirurid nematodes that have expanded their geographic distribution worldwide. They infect a variety of mammalian definitive hosts with few reports of potential paratenic infections in amphibian and reptile hosts. In this study, we report *Pterygodermatites* sp. larvae identified in free-ranging, invasive Cuban treefrogs (*Osteopilus septentrionalis*), from central Florida, United States. Encysted larvae were recovered from the skeletal muscle and/or the coelomic cavity of three frogs; molecular characterization of the small subunit (18S) ribosomal RNA and cytochrome oxidase I genes of the parasites matched reported sequences of *Pterygodermatites (Mesopectines) whartoni* (Tubangui, 1931). This is a parasite native to Southeastern Asia and to the best of the authors’ knowledge, it is the first report of the species in the New World. The recovery of invasive *Pterygodermatites* from invasive Cuban treefrogs in North America highlights the growing concern regarding the potential impact non-native parasites and invasive species may have on native wildlife populations.

## 1 Introduction

Species of *Pterygodermatites* are spirurid nematodes belonging to the family Rictulariidae. The adult worms inhabit the gastrointestinal tract of various mammals including rodents, cats, primates, and bats (1–5). Eggs are shed in the feces of the definitive host, which are ingested by an intermediate host, commonly an arthropod (3, 6). In the arthropod, larvae develop into an infective third stage (L3) and are transmitted to the definitive host following ingestion of the arthropod (3). There are few reports of this nematode in hosts other than mammals and arthropods (3). *Pterygodermatites* (*Multipectines*) *cahirensis* (Jägerskiöld, 1909) (syn. *Rictularia cahirensis*) has been reported in a species of gecko (*Hemidactylus flaviviridis*) and frog (*Hoplobatrachus tigerinus,* syn. *Rana tigrina*) in Asia as encysted or encapsulated larvae (3, 7). These larvae successfully infected a cat, demonstrating the potential of reptile and amphibian paratenic hosts in the life cycles of these nematodes (7), and that completion of the life cycle required two hosts, one an invertebrate and the second a lizard or snake (4).

Species of *Pterygodermatites* have expanded their geographic distribution worldwide, facilitating interactions with novel definitive, intermediate and paratenic hosts (3). Currently, there are reports of *Pterygodermatites* infections in mice and rats from Florida (8, 9), but none in humans or other primates. While there are currently no reports of amphibian infections in Florida, anuran diet and ecology allow for interactions with known intermediate and definitive hosts. Moreover, the spectrum of potential host amphibians in Florida is ever growing due to the establishment of invasive species that can serve as hosts for both native and invasive parasites. For instance, the invasive Cuban treefrog (*Osteopilus septentrionalis*) is documented as a potential paratenic and intermediate host for invasive *Angiostrongylus cantonensis* (10) and *Raillietiella orientalis* (11) respectively, in Florida. Both of these parasites may pose a threat to native Florida wildlife.

There is limited information regarding the relationship between *Pterygodermatites* spp. and reptile and amphibian paratenic hosts. As many of the invertebrates known to harbor *Pterygodermatites* (7, 9, 12) can be found in the diet of Cuban treefrogs (13), there is the potential that they may serve as intermediate or paratenic hosts in Florida. Species that predate known hosts of *Pterygodermatites* spp., including free-roaming domestic cats, also may predate Cuban treefrogs (14). While many species of *Pterygodermatites* have been shown to infect captive wildlife species in zoos (2, 15), free-ranging predator–prey dynamics outside of the zoo environment could allow for infections of novel intermediate and paratenic hosts.

This report documents three cases of natural *Pterygodermatites (Mesopectines) whartoni* (Tubangui, 1931) infection in invasive Cuban treefrogs (*O. septentrionalis*) in central Florida. This report demonstrates (i) Cuban treefrogs can serve as potential paratenic hosts of *P. whartoni*, (ii) *P. whartoni* is not restricted to southeastern Asia, and (iii) the role invasive species can play in invasive parasite distribution and transmission.

## 2 Materials and methods

**Figure 1:**
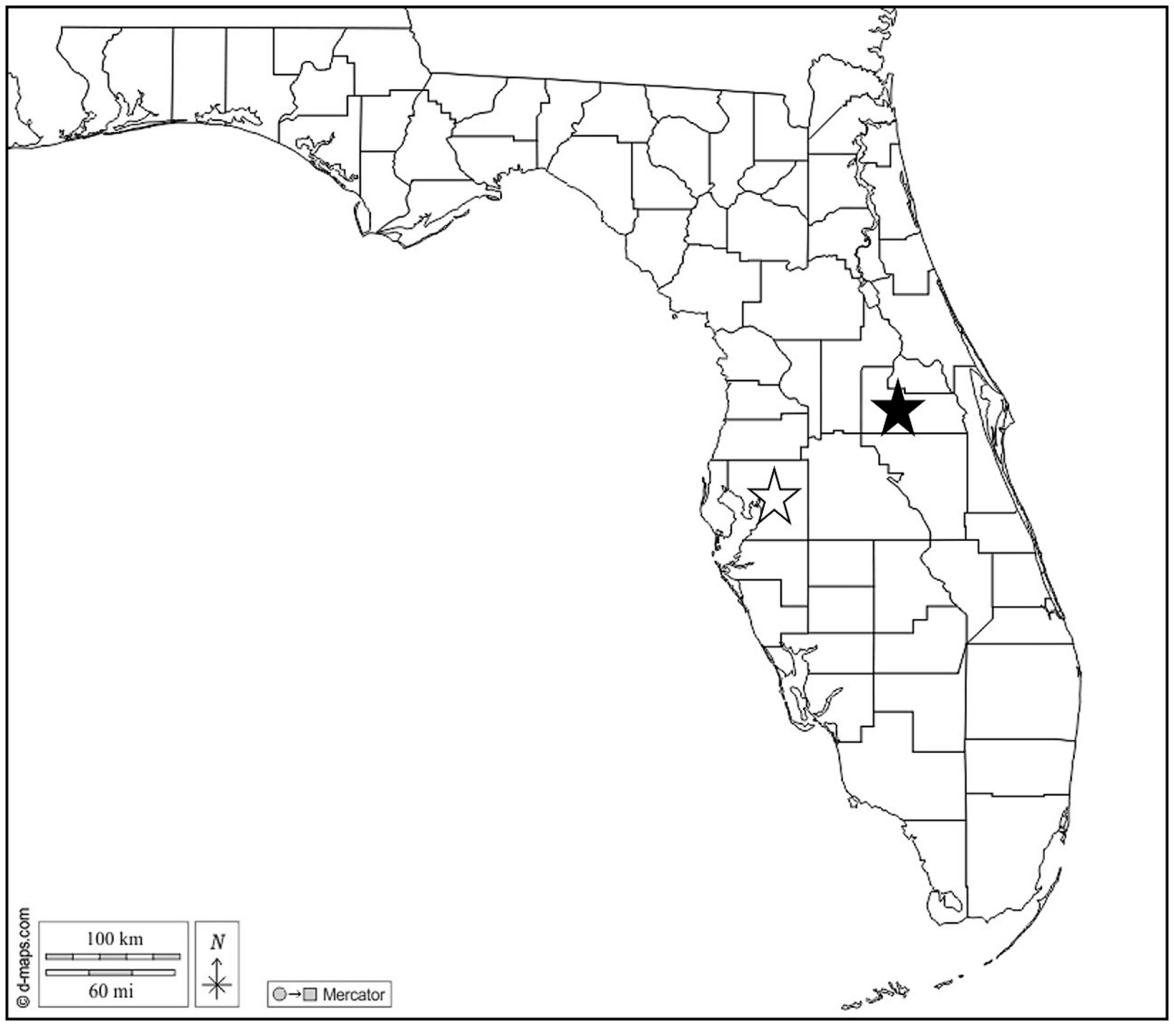
Map of Florida, US. Stars indicate Hillsborough (open) and Orange (solid) Counties where Cuban treefrogs were collected. Map courtesy of d-maps.com. https://d-maps.com/carte.php?num_car=20131&lang=en.

In June and July 2021, *O. septentrionalis* adults were collected from Hillsborough (*n* = 2 female, *n* = 8 male) and Orange (*n* = 6 female, *n* = 7 male) Counties in Florida, United States (Figure 1). Frogs were captured by hand from PVC-pipe refugia deployed in ornamental plant gardens at Hillsborough Community College (Plant City Campus) and the Orange County Extension office. This study was approved by the Institutional Animal Care and Use Committee at the University of Florida IACUC #202011222 and all frogs were collected and evaluated per approved protocols. Frogs were euthanized by immersion in buffered tricaine methanesulfonate (MS-222) prior to exposure of the coelomic cavity and submersed in 70% ethanol for preservation. Ethanol fixed liver, lung, hind leg muscle, and the full gastrointestinal tract tissues were removed and examined grossly and microscopically for parasites; samples of the brain, heart, liver, lung and hind leg skeletal muscle were processed for microscopic examination. Sections were cut at 5 μm, stained with hematoxylin and eosin (H&E), and examined histologically for the presence of parasites.

All nematodes were removed from tissue, transferred to 70% ethanol and examined microscopically for morphological identification. DNA was extracted from each nematode using the DNeasy Blood and Tissue Kit (Qiagen, Valencia, CA) per manufacturer’s recommendations.

Portions of both the small subunit (18S) ribosomal RNA [using primers 18SF-342 and 18S-530R as reported in Thomas (16)] and the cytochrome oxidase I [using primers COIintF and COIintR as reported in Hamer et al. (17)] were amplified from extracted nucleic acids as previously described. PCR products were visualized by gel electrophoresis, and amplicons of the correct size (approximately 480 and 690 base pairs [bp], respectively) were excised and gel extracted using the QIAquick Gel Extraction Kit (Qiagen, Valencia, CA) per manufacturer’s recommendations. Gel extracted amplicons were submitted for bidirectional commercial sequencing (Genewiz, Azenta Life Sciences). Sequences were assembled and edited using commercial bioinformatic software (Geneious 11.1.5; Biomatters, Auckland, New Zealand). The determined sequences were compared to known nematode sequences using the NCBI Basic Local Alignment Search Tool (18). Consensus 18S and COI gene sequences were uploaded to GenBank (OR861525 & OR861524, respectively).

## 3 Results

**Figure 2:**
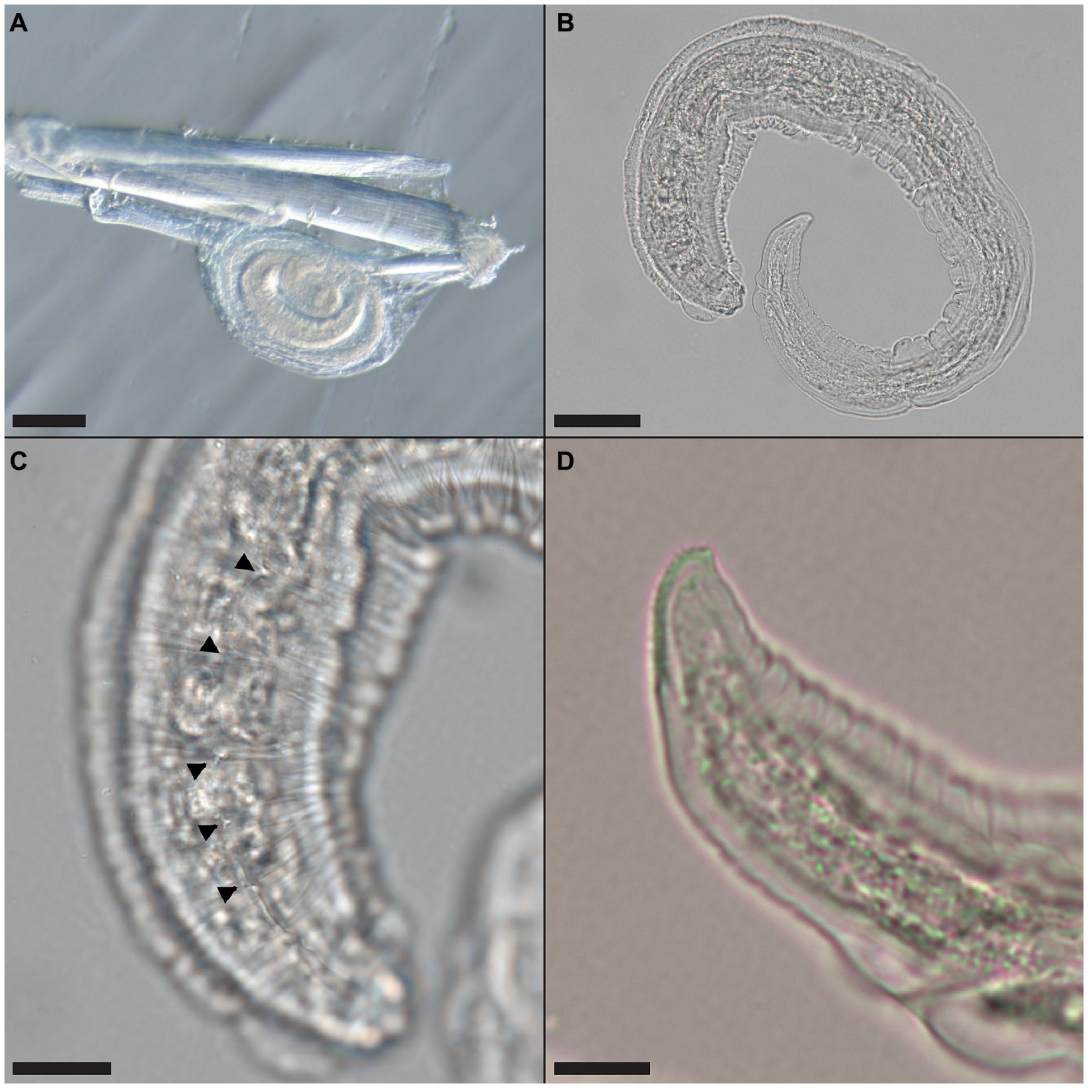
*Pterygodermatites whartoni* larvae recovered from free-ranging, invasive Cuban treefrog (*Osteopilus septentrionalis*) leg muscle. **(A)** Coiled larva attached to a muscle fiber; scale bar = 100 μm. **(B)** Larva removed from cyst capsule; scale bar = 50 μm. **(C)** Cuticular spines (arrowheads) along the cuticle of the larva, anterior end shown; scale bar = 20 μm. **(D)** Toothed or ridged posterior end of the larva; scale bar = 10 μm.

Multiple nematode larvae consistent with *Pterygodermatites* sp. were recovered encysted in the hind leg muscle of a male *O. septentrionalis* collected from Hillsborough County (Frog OS2021-59) and the leg muscle and adhered to the serosa outside of the stomach of two female *O. septentrionalis* collected from Orange County (Frogs OS2021-64, -65). The recovered nematodes were cleared with lactophenol and examined under a dissecting and compound microscope. The oval cysts contained a single, coiled larva and were approximately 285–288 μm in length (Figure 2A). Once removed from the cyst, the larva (Figure 2B) was approximately 580 μm in length and cuticular spines were visible from the anterior to midbody (Figure 2C); alae were also present. The tail tip had a distinct toothed or ridged end (Figure 2D).

No parasite was observed microscopically within the sections of hind leg skeletal muscle, heart, brain, liver, or lung of the three frogs noted to have nematode larvae. In two (OS2021-59 and -64) frogs, sections of nematodes were present within the liver, associated with a mild granulomatous reaction. In OS2021-59, nematode sections were characterized by a thick cuticle, coelomyarian-polymyarian musculature, prominent basophilic lateral cords, and portions of the intestinal tract lined by large, multinucleate enterocytes with abundant foamy cytoplasm and disorganized nuclei (data not shown). No sublateral alae were present. In OS2021-64, the nematodes in section were degenerate which precluded further characterization (data not shown).

Portions of the 18S rRNA and COI genes were amplified and sequenced directly from nematode larvae collected from the three Cuban tree frogs. After primer editing, a 447 bp fragment of the 18S gene was determined from each parasite. The nucleotide sequence was identical between the three parasites (Genbank OR861525), 100% identical to reported sequences of *P. whartoni* (MG489657, MG489658, MG489660), and 99.5% identical to *Pterygodermatites (Mesopectines) nycticebi* (Mönnig, 1920) (MG753548; Supplementary Table S1). For the COI gene, a 649 bp fragment was determined for each parasite; the nucleotide sequence between the three parasites was identical (Genbank OR861524). The parasite COI sequence exhibited 97.0 and 97.2% sequence identity to reference *P. whartoni* sequences (MZ476254 and MZ476257, respectively) and 88.8–89.1% identical to reference *P. nyctecebi* sequences (MZ476253, MG757149, and MZ476258; Supplementary Table S2).

## 4 Discussion

This report documents the presence of *Pterygodermatites* larvae in three invasive Cuban tree frogs (*Osteopilus septentrionalis*) in Florida. There are 68 species of *Pterygodermatites* described worldwide from mammalian hosts, with all known life cycles utilizing an arthropod intermediate host (3). No paratenic hosts have been described for *P. nycticebi* or *P. whartoni*; intermediate hosts have been suggested to be cockroaches or other arthropods (2, 3, 15). Confident determination of the species of *Pterygodermatites* in these frogs is limited by a general lack of molecular reference sequence for representatives of the genus. The NIH NCBI Nucleotide database contained only 28 *Pterygodermatites* spp. reference sequences at the time of analysis for this report, representing eight assigned and one unassigned species at three different loci: the cytochrome oxidase I (*n* = 15), small subunit (18S) rRNA (*n* = 11), and the large subunit rRNA (*n* = 2) gene. At the 18S gene, the larvae in these frogs were 100% identical to three sequences of *P. whartoni* reported from parasites collected from *Leopoldamys* giant rats in Viet Nam. However, at the COI gene, the frog parasites exhibited only 97.0–97.2% sequence identity to *P. whartoni* also from Viet Nam *Leopoldamys* giant rats. Unfortunately, no peer-reviewed publication is associated with the direct nucleotide submissions for *P. whartoni*, and no other genetic loci are published for additional comparison. When comparing reported COI sequences between different accessions of the same *Pterygodermatites* spp., differences of up to 3–4% can be seen (Supplementary Table S2; *Pterygodermatites (Paucipectines) jagerskioldi* (Lent & Freitas, 1935) [3.3%]; *Pterygodermatites (Paucipectines) zygodontomis* (Quentin, 1967) [2.8–3.5%]; *P. nycticebi* [1.4–3.9%]), though for the three published *P. whartoni* sequences, variation of only 0.5–0.9% is present. However, all of these sequences originate from the same research group studying *Leopoldamys* giant rats in Viet Nam, and as such may exhibit decreased sequence variation than parasites from more distant geographic locales. Given the 100% nucleotide identity at the 18S gene and COI nucleotide identity differences consistent with those observed for other *Pterygodermatites* species, the larvae found in these invasive frogs in Florida most likely represent *P. whartoni*.

Encysted larvae were identified within the limb skeletal muscle and the coelomic cavity of the frogs in this study. Microscopic examination of select tissues from the affected frogs did not reveal sections of identifiable *Pterygodermatites* spp. While two frogs had nematodes present in their livers, in one frog the degenerate nature of the parasites precluded identification; in the other frog, the features of the hepatic nematode, namely the absence of sublateral alae and the presence of multinucleate enterocytes in the intestinal tract, is inconsistent with previous reports of the histomorphology of *Pterygodermatites* (2). PCR was attempted on formalin fixed paraffin-embedded sections of the affected tissues, but either due to parasites being absent in deeper levels or limitations due to the limited parasite DNA in the extracts, molecular characterization of the hepatic parasites was not successful.

*Pterygodermatites nycticebi* and *P. whartoni* are found in primates and rodents, respectively (3). While primates infected with *P. nycticebi* have been documented with varying severity of disease that includes weakness, anemia and hypoproteinemia (15), little is known or documented regarding disease in rodents infected with *P. whartoni* (1, 19). *Pterygodermatites whartoni* has been recovered from rodents (*Rattus* spp., *Sundasciurus steerii juvencus*) in Taiwan, Japan and the Philippines (3, 19). Detection of encysted *P. whartoni* in Cuban treefrog tissues suggest that this amphibian is a potential paratenic host, much like the lizard *H. flaviviridis* and frog *H. tigerinus* have been described as paratenic hosts for *P. cahirensis* in cats (4, 7).

The Cuban treefrog is an invasive species in the US and has been documented throughout Florida and into the southeastern US (13). With a diet that includes a variety of insects (13), these frogs are well positioned to serve as paratenic hosts for species of *Pterygodermatites*. Cuban tree frogs are consumed by many species of snakes and birds, as well as free-roaming cats (13, 14). The dynamics of this life cycle and the use of human-associated paratenic hosts can help facilitate geographic spread and transmission of *Pterygodermatites* spp., as well as other pathogenic and potentially zoonotic parasites.

Additional research is needed to better characterize the larval stages of *Pterygodermatites* spp., and the hosts they utilize. The use of paratenic hosts allows the parasite to infect a wider variety of mammalian hosts, which may increase the likelihood of disease in new parasite–host interactions. Additionally, identifying *P. whartoni*, a parasite native to Asia, recovered from invasive Cuban treefrogs in North America highlights the growing concern regarding invasive parasites and their potential impact on native domestic and wildlife populations. As the movement of humans and animals continues to increase, introductions of invasive parasites will follow. Identifying potential health risks are important, as well as identifying ways to minimize transmission and limit the geographic spread of invasive parasites to safeguard vulnerable hosts.

## Data availability statement

The original contributions presented in the study are publicly available. This data can be found here: https://www.ncbi.nlm.nih.gov/; OR861525-OR861524.

## Ethics statement

The animal study was approved by Institutional Animal Care and Use Committee at the University of Florida. The study was conducted in accordance with the local legislation and institutional requirements.

## Author contributions

KL: Data curation, Investigation, Writing – original draft, Writing – review & editing. RO: Conceptualization, Data curation, Formal analysis, Funding acquisition, Investigation, Methodology, Resources, Writing – original draft, Writing – review & editing. EC: Data curation, Investigation, Writing – original draft, Writing – review & editing. NT: Data curation, Supervision, Writing – original draft, Writing – review & editing. TF: Data curation, Investigation, Methodology, Resources, Writing – original draft, Writing – review & editing. TW: Formal analysis, Writing – original draft, Writing – review & editing. SJ: Data curation, Investigation, Methodology, Resources, Writing – original draft, Writing – review & editing. HW: Conceptualization, Data curation, Formal analysis, Funding acquisition, Investigation, Methodology, Project administration, Resources, Supervision, Writing – original draft, Writing – review & editing.
