# Supplementary material for: Pterygodermatites (Mesopectines) whartoni (Nematoda: Rictulariidae) encysted larvae in invasive Cuban treefrogs (Osteopilus septentrionalis) from Florida, United States
Source: Front Vet Sci. 2024 May 10;11:1353975. doi: 10.3389/fvets.2024.1353975 (PMC11117167; doi:10.3389/fvets.2024.1353975)
Supplement: Supplementary file 1 [file Table_1.docx]

# Table 1. *Pterygodermatites* 18S gene fragment nucleotide identity distance table.

|  | *P. tani*  MT135073 | *P. tani*  MT135074 | *P. jagerskioldi*  KT894813 | *P. zygodontomis*  MK084860 | *P.* sp. DG-2021  MZ489659 | *P. whartoni*  MZ489660 | *P. whartoni*  MZ489658 | *P. whartoni*  MZ489657 | *P. nyctecebi*  MZ489661 | *P. nyctecebi*  MZ489656 | *P. nyctecebi*  MG753548 | **Cuban Tree Frog**  ***Pterygodermatites*** |
| --- | --- | --- | --- | --- | --- | --- | --- | --- | --- | --- | --- | --- |
| *P. tani*  MT135073 |  | 100 | 28 | 27.9 | 28.4 | 28.1 | 28.1 | 28.1 | 28.3 | 28.3 | 28.3 | 19.2 |
| *P. tani*  MT135074 | 100 |  | 28.1 | 28 | 28.6 | 28.3 | 28.3 | 28.3 | 28.4 | 28.4 | 28.4 | 19.4 |
| *P. jagerskioldi*  KT894813 | 28 | 28.1 |  | 98.2 | 92.1 | 91.8 | 91.8 | 91.8 | 91.8 | 91.9 | 92.4 | 93.5 |
| *P. zygodontomis*  MK084860 | 27.9 | 28 | 98.2 |  | 92 | 91.4 | 91.4 | 91.4 | 91.7 | 91.8 | 92.1 | 93.9 |
| *P.* sp. DG-2021  MZ489659 | 28.4 | 28.6 | 92.1 | 92 |  | 99.3 | 99.3 | 99.3 | 99.5 | 99.5 | 99.6 | 99.5 |
| *P. whartoni*  MZ489660 | 28.1 | 28.3 | 91.8 | 91.4 | 99.3 |  | 100 | 100 | 99.5 | 99.4 | 99.5 | 100 |
| *P. whartoni*  MZ489658 | 28.1 | 28.3 | 91.8 | 91.4 | 99.3 | 100 |  | 100 | 99.5 | 99.4 | 99.5 | 100 |
| *P. whartoni*  MZ489657 | 28.1 | 28.3 | 91.8 | 91.4 | 99.3 | 100 | 100 |  | 99.5 | 99.4 | 99.5 | 100 |
| *P. nyctecebi*  MZ489661 | 28.3 | 28.4 | 91.8 | 91.7 | 99.5 | 99.5 | 99.5 | 99.5 |  | 99.8 | 99.9 | 99.5 |
| *P. nyctecebi*  MZ489656 | 28.3 | 28.4 | 91.9 | 91.8 | 99.5 | 99.4 | 99.4 | 99.4 | 99.8 |  | 99.9 | 99.5 |
| *P. nyctecebi*  MG753548 | 28.3 | 28.4 | 92.4 | 92.1 | 99.6 | 99.5 | 99.5 | 99.5 | 99.9 | 99.9 |  | 99.5 |
| **Cuban Tree Frog**  ***Pterygodermatites*** | 19.2 | 19.4 | 93.5 | 93.9 | 99.5 | 100 | 100 | 100 | 99.5 | 99.5 | 99.5 |  |
