# Supplementary material for: Pterygodermatites (Mesopectines) whartoni (Nematoda: Rictulariidae) encysted larvae in invasive Cuban treefrogs (Osteopilus septentrionalis) from Florida, United States
Source: Front Vet Sci. 2024 May 10;11:1353975. doi: 10.3389/fvets.2024.1353975 (PMC11117167; doi:10.3389/fvets.2024.1353975)
Supplement: Supplementary file 2 [file Table_2.docx]

# Table 2. *Pterygodermatites* COI gene fragment nucleotide identity distance table. Black boxes highlight multiple accessions for the same species.

|  | *P.* sp. DG-2021  MZ476256 | *P. valladaresi*  OP086098 | *P. plagiostoma*  ON502379 | *P. jagerskioldi*  KT894802 | *P. jagerskioldi*  MF155935 | *P. zygodontomis*  MF187069 | *P. zygodontomis*  MF155934 | *P. zygodontomis*  MF155933 | *P. nyctecebi*  MZ476258 | *P. nyctecebi*  MG757149 | *P. nyctecebi*  MZ476253 | *P. whartoni*  MZ476255 | *P. whartoni*  MZ476254 | *P. whartoni*  MZ476257 | **Cuban Tree Frog**  ***Pterygodermatites*** |
| --- | --- | --- | --- | --- | --- | --- | --- | --- | --- | --- | --- | --- | --- | --- | --- |
| *P.* sp. DG-2021  MZ476256 |  | 90.4 | 74.3 | 71.7 | 71.4 | 70.4 | 71.3 | 71.7 | 90.5 | 90.4 | 90.7 | 89.8 | 89.8 | 89.6 | 90 |
| *P. valladaresi*  OP086098 | 90.4 |  | 74.7 | 71.7 | 72.3 | 70.2 | 70.6 | 70 | 87.4 | 87.4 | 87.4 | 90.7 | 90.4 | 90.4 | 89.3 |
| *P. plagiostoma*  ON502379 | 74.3 | 74.7 |  | 78.6 | 80.1 | 76.1 | 76.2 | 76 | 74.3 | 74.9 | 75.4 | 73.5 | 73.8 | 73.5 | 73.7 |
| *P. jagerskioldi*  KT894802 | 71.7 | 71.7 | 78.6 |  | 96.7 | 84.6 | 84.4 | 84.1 | 74 | 72.2 | 73.3 | 72.2 | 72.2 | 71.7 | 72.2 |
| *P. jagerskioldi*  MF155935 | 71.4 | 72.3 | 80.1 | 96.7 |  | 85.3 | 84.9 | 84.4 | 72.1 | 70.7 | 71.8 | 71.1 | 71.1 | 70.7 | 70.8 |
| *P. zygodontomis*  MF187069 | 70.4 | 70.2 | 76.1 | 84.6 | 85.3 |  | 97.2 | 96.5 | 73 | 73.9 | 73.9 | 72.8 | 72.6 | 72.8 | 71.8 |
| *P. zygodontomis*  MF155934 | 71.3 | 70.6 | 76.2 | 84.4 | 84.9 | 97.2 |  | 97.2 | 73.3 | 74.2 | 74.2 | 72.9 | 72.7 | 72.9 | 72.5 |
| *P. zygodontomis*  MF155933 | 71.7 | 70 | 76 | 84.1 | 84.4 | 96.5 | 97.2 |  | 74.1 | 74.6 | 74.6 | 73 | 72.8 | 73 | 72.8 |
| *P. nyctecebi*  MZ476258 | 90.5 | 87.4 | 74.3 | 74 | 72.1 | 73 | 73.3 | 74.1 |  | 96.1 | 96.5 | 88.4 | 88.6 | 88.8 | 89.1 |
| *P. nyctecebi*  MG757149 | 90.4 | 87.4 | 74.9 | 72.2 | 70.7 | 73.9 | 74.2 | 74.6 | 96.1 |  | 98.6 | 88.4 | 88.6 | 88.8 | 88.9 |
| *P. nyctecebi*  MZ476253 | 90.7 | 87.4 | 75.4 | 73.3 | 71.8 | 73.9 | 74.2 | 74.6 | 96.5 | 98.6 |  | 88.2 | 88.4 | 88.2 | 88.8 |
| *P. whartoni*  MZ476255 | 89.8 | 90.7 | 73.5 | 72.2 | 71.1 | 72.8 | 72.9 | 73 | 88.4 | 88.4 | 88.2 |  | 99.5 | 99.3 | 97 |
| *P. whartoni*  MZ476254 | 89.8 | 90.4 | 73.8 | 72.2 | 71.1 | 72.6 | 72.7 | 72.8 | 88.6 | 88.6 | 88.4 | 99.5 |  | 99.1 | 96.8 |
| *P. whartoni*  MZ476257 | 89.6 | 90.4 | 73.5 | 71.7 | 70.7 | 72.8 | 72.9 | 73 | 88.8 | 88.8 | 88.2 | 99.3 | 99.1 |  | 97 |
| **Cuban Tree Frog**  ***Pterygodermatites*** | 90 | 89.3 | 73.7 | 72.2 | 70.8 | 71.8 | 72.5 | 72.8 | 89.1 | 88.9 | 88.8 | 97 | 96.8 | 97 |  |
